# Identification of Putative Chemosensory Receptor Genes from the *Athetis dissimilis* Antennal Transcriptome

**DOI:** 10.1371/journal.pone.0147768

**Published:** 2016-01-26

**Authors:** Junfeng Dong, Yueqin Song, Wenliang Li, Jie Shi, Zhenying Wang

**Affiliations:** 1 Forestry College, Henan University of Science and Technology, Luoyang, 471003, China; 2 Institute of Plant Protection, Hebei Academy of Agricultural and Forestry Sciences, Baoding, 071000, China; 3 State Key Laboratory for the Biology of the Plant Diseases and Insect Pests, Institute of Plant Protection, Chinese Academy of Agricultural Sciences, Beijing, China; Institute of Zoology, CHINA

## Abstract

Olfaction plays a crucial role in insect population survival and reproduction. Identification of the genes associated with the olfactory system, without the doubt will promote studying the insect chemical communication system. In this study, RNA-seq technology was used to sequence the antennae transcriptome of *Athetis dissimilis*, an emerging crop pest in China with limited genomic information, with the purpose of identifying the gene set involved in olfactory recognition. Analysis of the transcriptome of female and male antennae generated 13.74 Gb clean reads in total from which 98,001 unigenes were assembled, and 25,930 unigenes were annotated. Total of 60 olfactory receptors (ORs), 18 gustatory receptors (GRs), and 12 ionotropic receptors (IRs) were identified by Blast and sequence similarity analyzes. One obligated olfactory receptor co-receptor (Orco) and four conserved sex pheromone receptors (PRs) were annotated in 60 ORs. Among the putative GRs, five genes (AdisGR1, 6, 7, 8 and 94) clustered in the sugar receptor family, and two genes (AdisGR3 and 93) involved in CO_2_ detection were identified. Finally, AdisIR8a.1 and AdisIR8a.2 co-receptors were identified in the group of candidate IRs. Furthermore, expression levels of these chemosensory receptor genes in female and male antennae were analyzed by mapping the Illumina reads.

## Introduction

*Athetis dissimilis* (Hampson, 1909) (Lepidoptera: Noctuidae) is found in many countries including Japan, Korea, India, Philippines and Indonesia [1–4]. In 2012, it was first observed that this species caused damage to summer maize seedling in Shandong province in China, although it had not been documented previously as an agricultural pest [4]. Since then, this pest has been found in Henan, Shanxi and Anhui provinces. Because of the fact that larvae of *A*. *dissimilis* live under plant residues, it is difficult to control the spread of the pest with chemical pesticides. Therefore, novel control strategies are urgently needed to mitigate crop damage.

Olfaction plays several vital roles in insect biology, including food selection, mate choice, the location of suitable oviposition sites by females, warning, and defense [5]. Accurate detection of volatile compounds in the surrounding environment is essential for insect survival. Antennae are specialized the main olfactory organs containing a large variety of sensilla. Environmental chemical compounds transported from micro-pore on the sensilla through antennal lymph to olfactory receptor neurons (ORNs) that generate an electrical impulse [6]. Several families of transmembrane proteins at the membrane surface of ORNs appear to detect and recognize odorant molecules [7]. These transmembrane protein families occupied with odorant molecules classified as olfactory receptors (ORs), gustatory receptors (GRs), and ionotropic receptors (IRs) [8–12]. Insect OR proteins contain seven transmembrane domains, but they have an inverted topology compared to those of vertebrates [13,14]. To function, one conventional OR and one obligate olfactory co-receptor (Orco) must form a dimer complex that works as a ligand-gated ion channel [13,15–17]. ORs in moths contain pheromone receptors (PRs) detecting sex pheromone and non-PR ORs. GRs were mainly expressed in the gustatory organs such as the mouthparts [18], in fact, some GRs are also expressed in olfactory structures and presumably have olfactory function [19]. The conservation of GR sequences is much higher than the ORs [20,21]. IRs is another variant subfamily of ionotropic glutamate receptors (iGluRs) [13]. In insects, the IR family includes the conserved “antennal IRs” having an olfactory function, and the species-specific “divergent IRs” having gustatory function [22].

The identification of chemosensory receptor genes in pest insects is especially significant due to their potential as novel targets in insect pest control. With the improvement of high-throughput sequencing methods, more chemosensory receptors have been discovered to date. Transcriptome sequencing or RNA sequencing (RNA-seq) is one common method that helps to obtain a large variety of functional genes. It has been used widely to identify genes involved in chemosensation in insects [23,24].

In order to identify chemosensory receptor genes of *A*. *dissimilis*, an organism with no available genomic information, we sequenced and analyzed an antennae transcriptome of adult females and males using Illumina HiSeq2500 sequencing. We report here that the antennal transcriptome of *A*. *dissimilis* includes 60 OR, 18 GR and 12 IR genes.

## Materials and Methods

### Insect rearing and antennae collection

*Athetis dissimilis* originally collected in July 2012 from infested maize seedlings in the Experiment Station of Henan University of Science and Technology in Luoyang, Henan province, China. The insects were fed with an artificial diet in the laboratory under conditions of 27 ± 1°C with 70 ± 5% relative humidity and maintaining 16 h: 8 h light/dark cycle. After pupation, pupae sexed according to the position of the genital scar. Male and female pupae were stored in separate cages for the emergence. Adults fed with 10% sugar solution. About 200 pairs of antennae of 3–4 days old male and female moths were excised and immediately stored in liquid nitrogen until use.

### RNA purification and sequencing

Total RNA was extracted using the RNAiso Plus kit (TaKaRa) and treated with RNase-free DNase I (TaKaRa) to remove residual DNA following the manufacturer's instructions and then measured for purity, concentration and integrity respectively using NanoDrop 2000c spectrophotometer (NanoDrop Products, Thermo Scientific, USA), Qubit 2.0 (Qubit^®^ 2.0 fluorometer, Life Technologies, USA) and Agilent 2100 (Quantifluor-ST fluorometer, Promega, USA). The qualified RNA samples were then used for transcriptome sequencing.

Following the TruSeq RNA Sample Preparation Guide v2 (Illumina), mRNA was enriched using oligo (dT) magnetic beads and sheared to create short fragments by adding Fragmentation Buffer. The first strand cDNAs were synthesized using random hexamer primers, which were further transformed into double stranded cDNA by using dNTPs, RNase H and DNA polymerase I. Next to the purification of the double stranded cDNA with AMPure XP beads, the end-repairing, Poly-A tailing and, sequencing adapters linking processes were completed. The size of the fragment was chosen using AMPure XP beads, and cDNA library was constructed by PCR amplification (Veriti^®^ 96-Well Thermal Cycle, Applied Biosystems, USA). The concentration and insert size of cDNA library were detected using Qubit 2.0 and Agilent 2100, and quantified with q-PCR (CFX-96, Bio-Rad, USA). Finally, 125 bp pair-end reads were generated by sequencing cDNA with Illumina HiSeq2500 based on sequencing-by-synthesis method. Sequencing analysis was performed by the Genomics Services Lab of Beijing Biomarker Technologies Co., Ltd. (Beijing, China). The raw data processing and base calling were performed by the Illumina instrument software.

### Unigene generation and annotation

In order to obtain the clean data, the raw reads were initially processed for removing the adapter sequences and low-quality bases. Then, the Q30 and GC-content were used to assess the sequencing quality. Sequenced reads were assembled de novo with Trinity software [25] by setting min_kmer_cov to 2 and all other parameters to default. Unigene sequences were aligned by online BLASTX program on the databases of NR, Swiss-Prot, KOG and KEGG using a cut-off E-value of 10^−5^. Unigenes were then annotated using BLAST with E-value of 10^−5^ and HMMER with E-value of 10^−10^. Then, NR BLASTX results were directed into GO annotation using Blast2GO. Genes are described in terms related to molecular function, cellular component or biological process. TransDecoder software was used to predict the coding sequences (CDS) and amino acid sequences of Unigene.

### Identification of the target genes and phylogenetic analyzes

Target sequences were identified from the BLAST results obtained by running against the database with E-value of < 10^−5^. The complete coding region was determined using the ORF finder (http://www.ncbi.nlm.nih.gov/gorf/gorf.html). The nucleotide sequences of annotated genes were translated into amino acid sequences using ExPASy portal (http://web.expasy.org/translate/). The transmembrane-domains (TMDs) of annotated genes were then predicted using TMHMM Server v. 2.0 (http://www.cbs.dtu.dk/services/TMHMM-2.0/). Genes of other insect species such as *Bombyx mori*, *Cydia pomonella*, and *Heliothis virescens* were used as references.

After completing the alignments of the candidate ORs, GRs and IRs using ClustalX (1.83) [26], phylogenetic trees were constructed using PhyML and Seaview v.4. The OR data set contains 204 genes in total, containing 60 candidate AdisOR sequences from *A*. *dissimilis*, 18 sequences from *Helicoverpa armigera* [7], 50 sequences from *B*. *mori* [27], 41 sequences from *C*. *pomonella* [23] and 35 sequences from *Danaus plexippus*. The GR data set, on the other hand, comprises respectively 17, 33 and 57 sequences from *Helicoverpa assulta* [28], *B*. *mori* [29], *Drosophila melanogaster* [30] in addition to 18 candidate AdisGR genes identified. In the IR data set, following numbers of sequences were collected 11 IRs from *Spodoptera littoralis* [31], 31 IRs from *H*. *assulta* [28], 6 IRs from *Tenebrio molitor* [32], 15 IRs from *Dendroctonus ponderosae* [33], and 7 IRs from *Ips typographus* [33]. The phylogenetic trees were viewed and edited using FIG TREE v. 1.3.1.

### OR, GR and IR transcription abundance analysis

Transcription levels of OR, IR and GR genes of *A*. *dissimilis* are reported in values of Fragments Per Kilobase of transcript per million mapped reads (FPKM). The FPKM measure considers the effect of sequencing depth and gene length for the read count at the same time, and is currently the most commonly used method for estimating gene expression levels [34]. Thus, the FPKM of each gene was calculated based on the length of the gene and read count mapped to this gene.

## Results

### Sequence analysis and assembly

We obtained 26,234,196 female and 28,315,769 male clean reads with a total of 13.74 Gb nucleotides from the antennal cDNA libraries. The sample GC content was consistently about 45%, and the average quality value was ≥ 30 for more than 87.83% of the cycle (**Table 1**). In total 10,821,996 contigs were generated with a k-mer of 25. Then 177,477 transcripts and 98,001 unigenes with N50 length of 1,666 and 1,172, were obtained from assembled using Trinity (**Table 2**).

**Table 1:** Summary of Illumina transcriptome sequencing for *Athetis dissimilis*.

Samples	Read Number	Base Number	GC Countent	%≥Q30
♀ antennae	26,234,196	6,607,416,977	45.92%	87.83%
♂ antennae	28,315,769	7,132,586,940	45.17%	88.32%

**Table 2:** Summary of de novo assembly of the *Athetis dissimilis* transcriptome.

Length Range	Contig	Transcript	Unigene
200–300	10,773,766 (99.55%)	69,750 (39.30%)	55,927 (57.07%)
300–500	22,814 (0.21%)	33,897 (19.10%)	18,694 (19.08%)
500–1000	11,939 (0.11%)	30,000 (16.90%)	10,148 (10.35%)
1000–2000	7,405 (0.07%)	25,057 (14.12%)	7,329 (7.48%)
2000+	6,072 (0.06%)	18,773 (10.58%)	5,903 (6.02%)
Total Number	10,821,996	177,477	98,001
Total Length	538,597,162	149,497,716	58,127,312
N50 Length	49	1,644	1,172
Mean Length	49.77	842.35	593.13

### Sequence annotation

**Fig 1:**
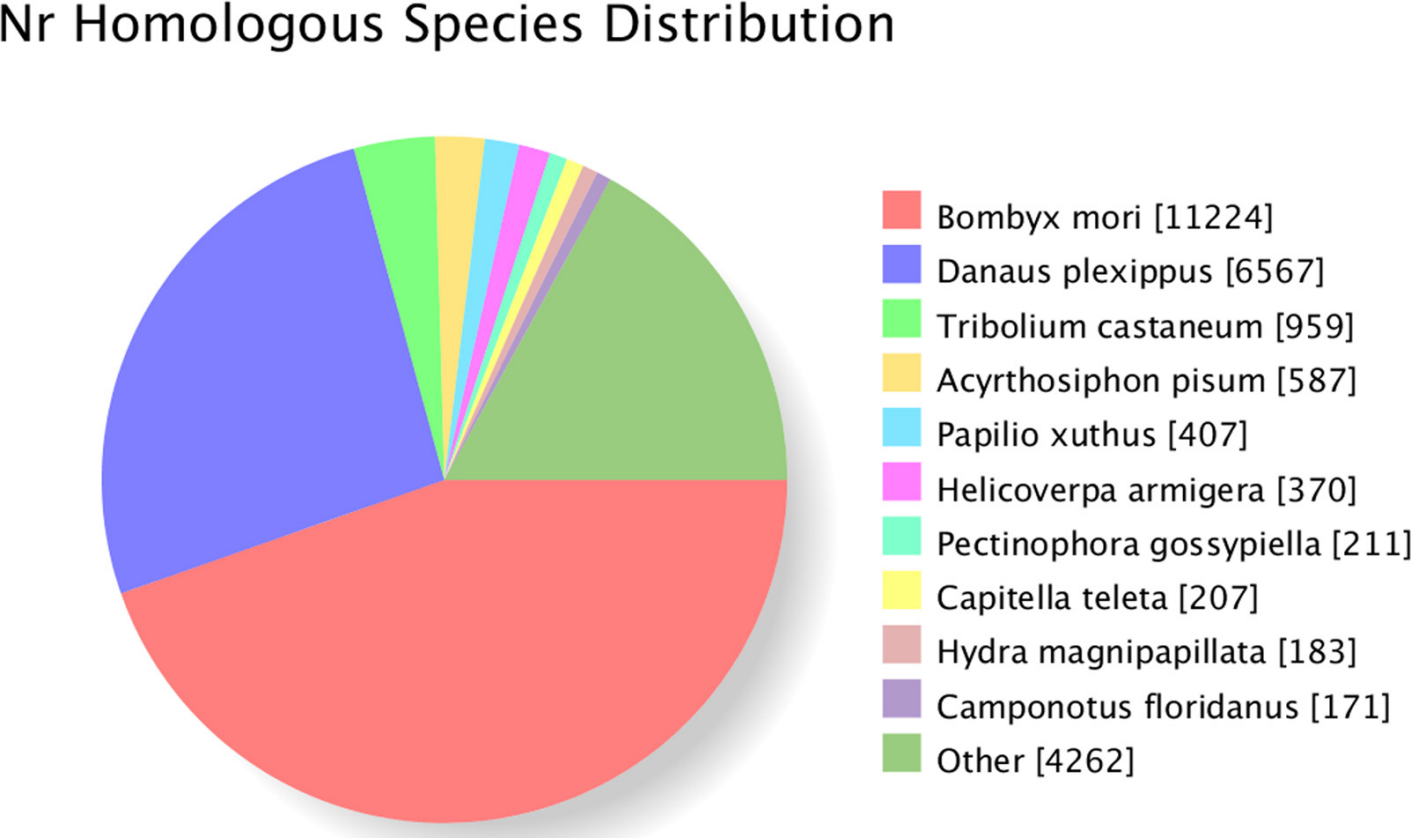
Characteristics of homology search for *Athetis dissimilis* unigenes. The number of unigenes matching the top ten species using BlastX in the Nr database is indicated in square brackets

The unigenes were annotated according to the alignments done in the diverse protein databases listed in the Methods section. The analyze showed significant matches of 25,355 unigenes (25.87%) in the NR, 14,618 unigenes (14.92%) in the Pfam, 14,026 unigenes (14.92%) in the KOG, and 13,807 unigenes (14.09%) in the Swiss-Prot databases. As a result, up to 25,930 putative coding sequences were identified (**Table 3**). NR database queries revealed that a high percentage of *A*. *dissimilis* sequences closely matched to sequences of *B*. *mori* (11224, 44.63%), followed by *D*. *plexippus* (6567, 26.11%), *Tribolium castaneum* (959, 3.70%), *Acyrthosiphon pisum* (587, 2.26%), and *Papilio xuthus* (407, 1.57%) respectively (**Fig 1**).

**Table 3:** Functional annotation of the *Athetis dissimilis* transcriptome.

Annotated databases	All sequences	≥ 300 bp	≤ 1000 bp
COG_Annotation	5967	1537	3722
GO_Annotation	9170	3089	3930
KEGG_Annotation	5678	1618	3206
KOG_Annotation	14026	4191	7657
Pfam_Annotation	14618	4416	8420
Swiss-Prot_Annotation	13807	4067	7800
nr_Annotation	25355	9420	10457
All_Annotated	25930	9675	10487

**Fig 2:**
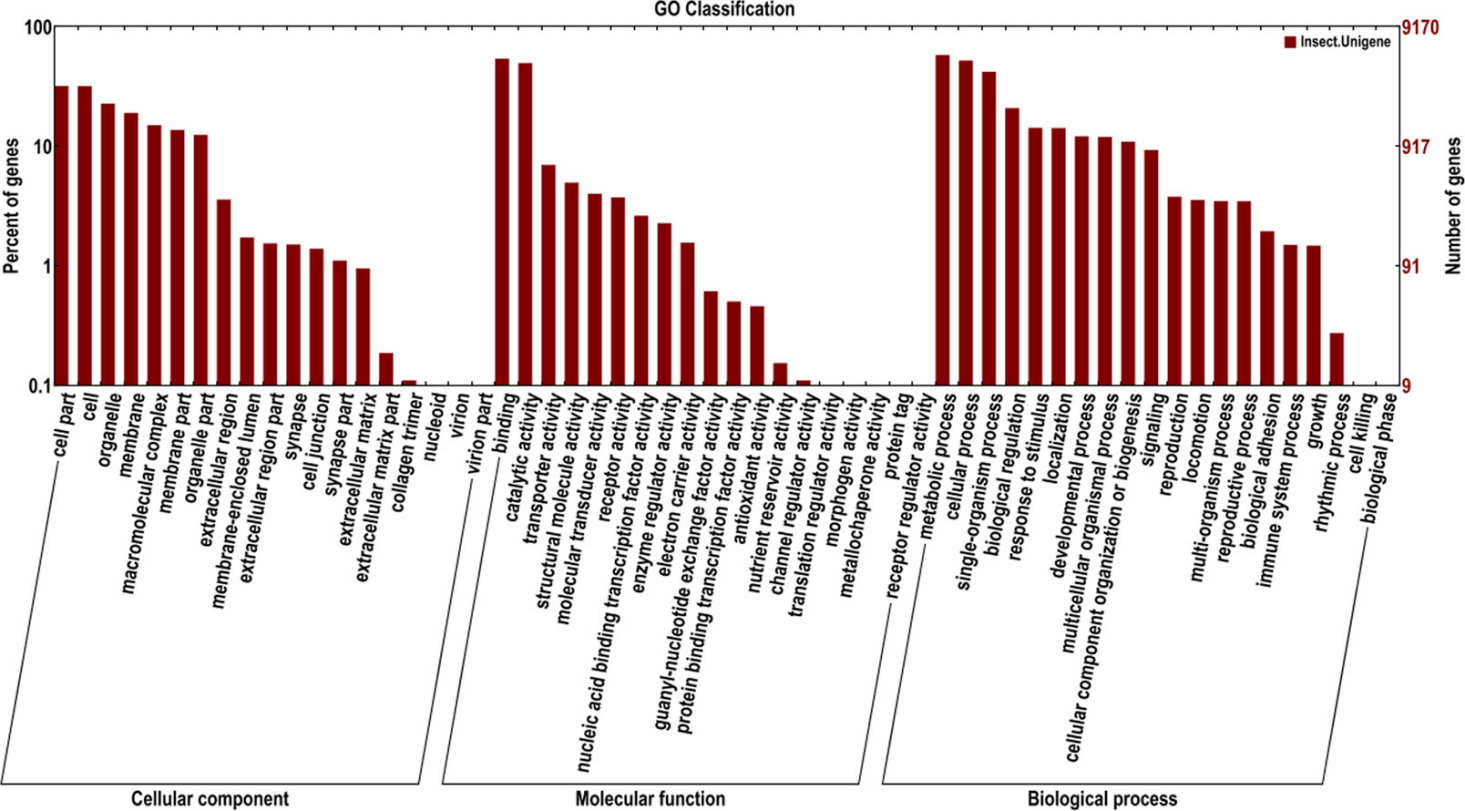
Functional annotation of assembled sequences based on gene ontology (GO) categorization. GO analysis was performed at the level for three main categories (cellular component, molecular function, and biological process)

**Fig 3:**
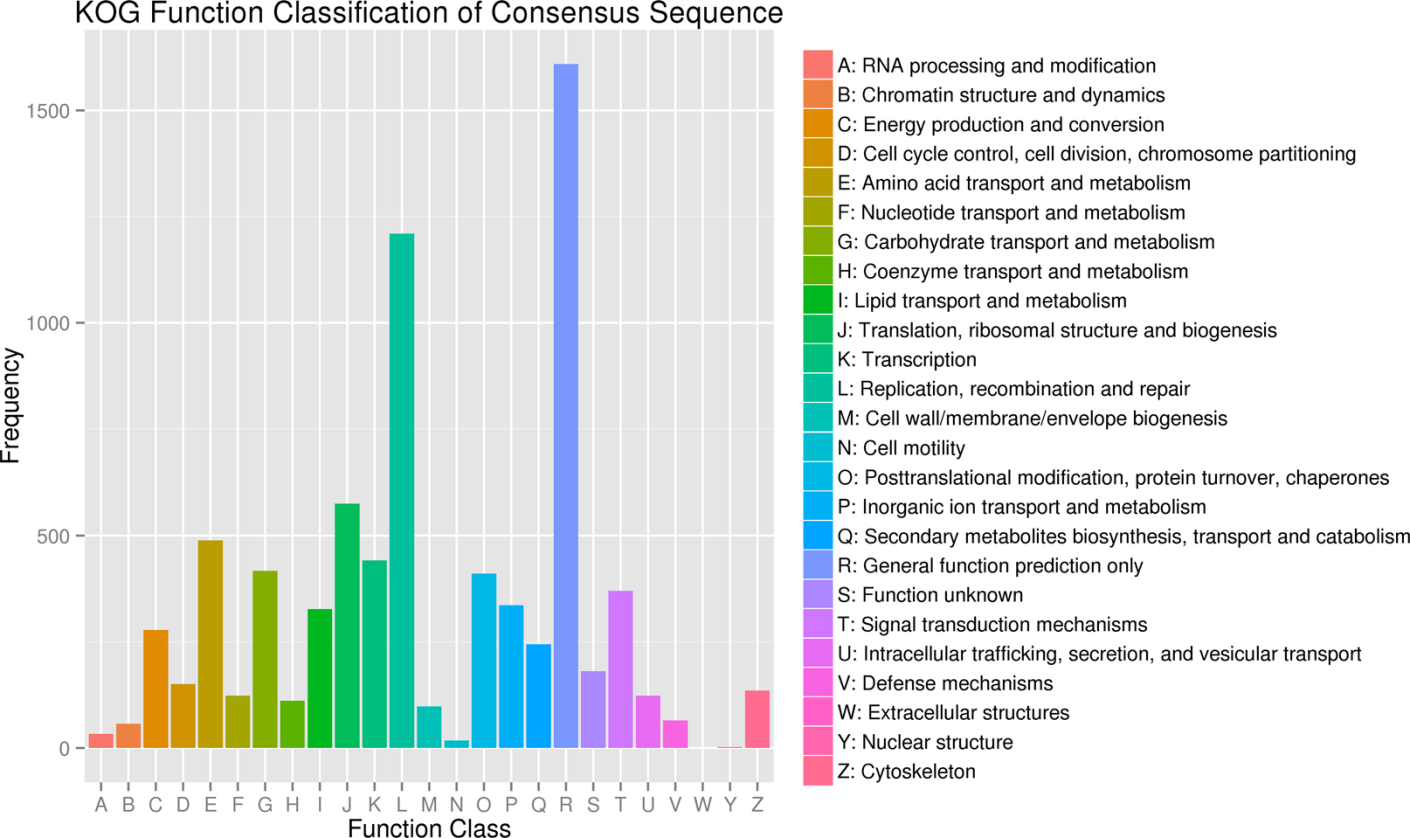
Clusters of orthologous groups (COG) classification. In total, 5967 unigenes with Nr hits were grouped into 25 COG classifications

**Fig 4:**
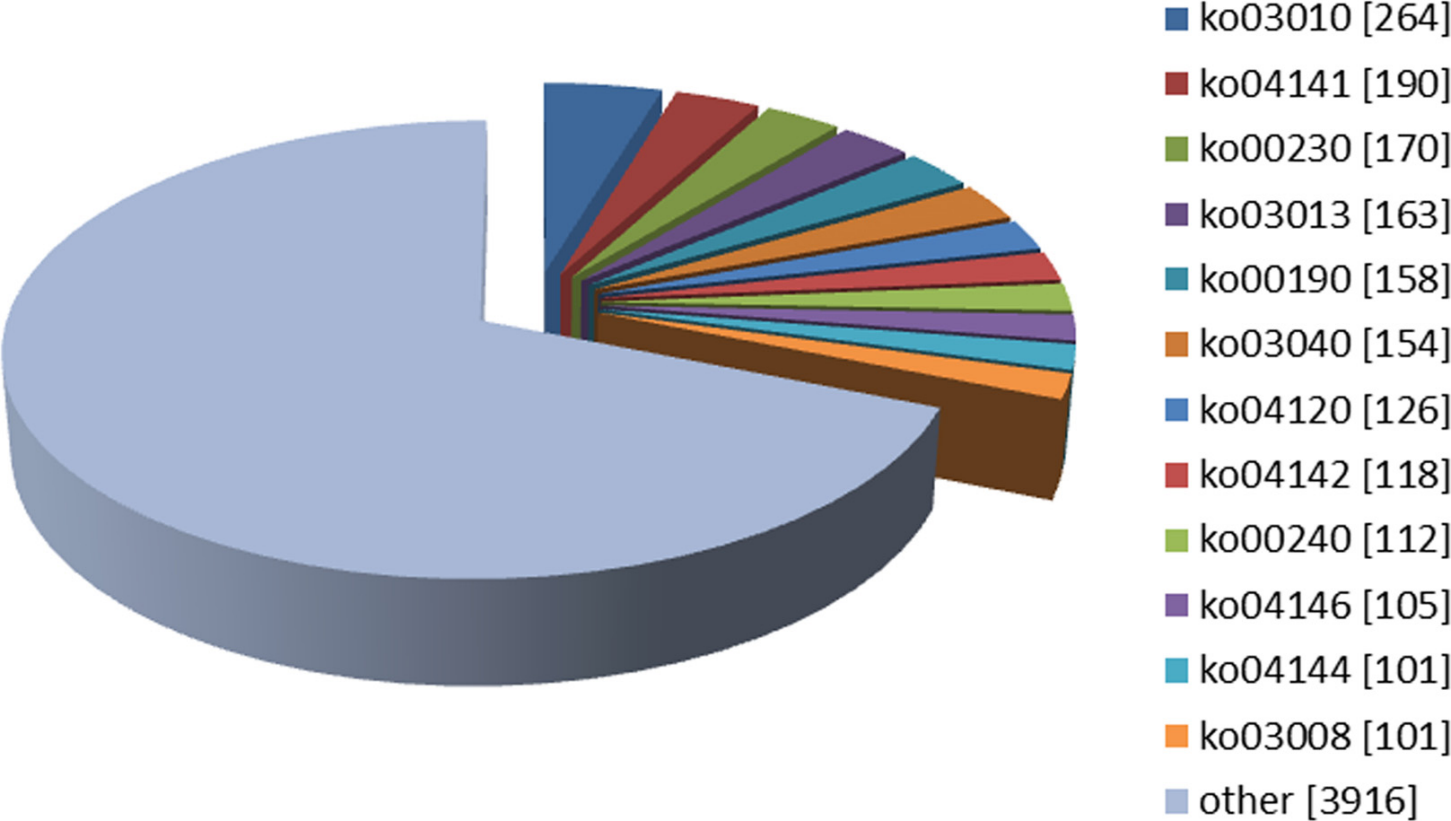
Distribution of each KEGG pathway number against the KEGG database. Each color represents a KEGG pathway. The top 13 KEGG pathways are indicated. The number of unigenes mapped in each pathway is indicated in square brackets. The abbreviations represent the pathways as follows: Ko03010: Ribosome; ko04141: Protein processing in endoplasmic reticulum; ko00230: Purine metabolism; ko03013: RNA transport; ko00190: Oxidative phosphorylation; ko03040: Spliceosome; ko04120: Ubiquitin mediated proteolysis; ko04142: Lysosome; ko00240: Pyrimidine metabolism; ko04146: Peroxisome; ko04144: Endocytosis; ko03008: Ribosome biogenesis in eukaryotes.

In a total, 38,759 unigenes were classified into following three ontologies with the GO analysis: i.) Molecular Function: 14,727 unigenes (38.00%), ii.) Cellular Components: 6,512 unigenes (16.80%) and, iii.) Biological Processes: 17,520 unigenes (45.20%) (**Fig 2**). Especially the proteins involved in binding in the Molecular Function category were abundant, which enabled us to identify the genes related to the olfactory recognition pathways. In addition to this, all unigenes were searched against the COG database for functional prediction and classification. After all, they were grouped into 25 specific categories (**Fig 3**). The largest group was “General function prediction only” (1609 genes, 26.97%) succeeding with “replication, recombination and repair” (1210, 20.28%), “translation, ribosomal structure and biogenesis” (575, 9.64%), “amino acid transport and metabolism” (489, 8.20%), “carbohydrate transport and metabolism” (417, 6.99%), “post-translation modification, protein turnover and chaperones” (411, 6.89%), and “signal transduction mechanisms” (370, 6.20%) which is one of the most important categories assigned for insect chemical signal transduction. Only a few unigenes were assigned to the functional groups like “cell motility” (18; 0.30%) and “nuclear structure” (2; 0.03%). In order to identify the biological pathways comprising annotated genes, 98,001 unigenes were mapped to reference canonical pathways in KEGG and correspondingly 5,678 sequences assigned into 184 KEGG pathways. The top 13 KEGG pathways contained over 100 unigenes (**Fig 4**). For example, 264 sequences belonged to the class “ribosome” (PATH: ko03010), followed by 190 in the “protein processing in endoplasmic reticulum” (PATH: ko04141) and 170 in “purine metabolism” (PATH: ko00230).

### Chemosensory receptors

**Fig 5:**
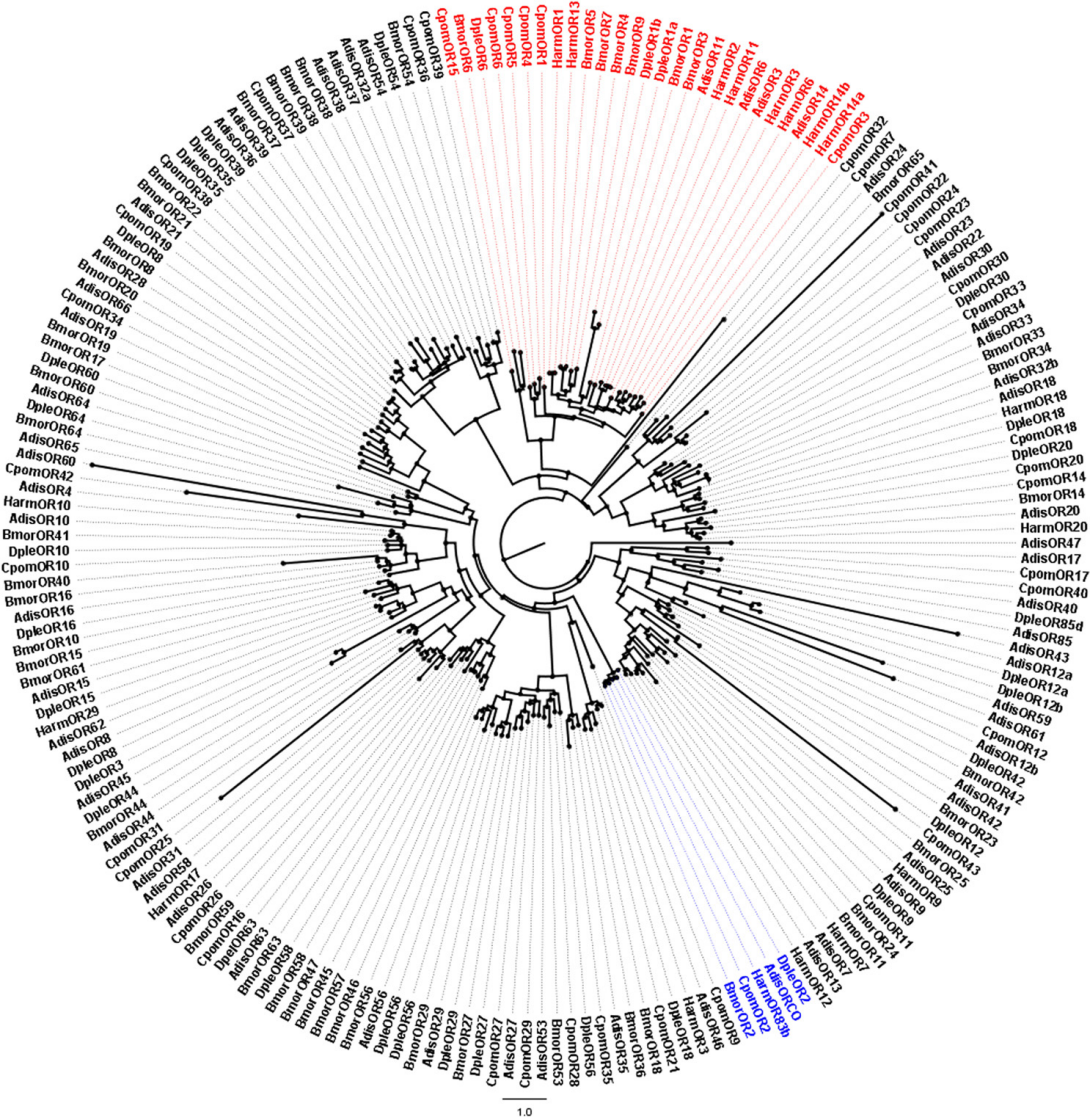
Neighbor-joining tree of candidate olfactory receptor (OR) and pheromone receptor (PR) genes from *Athetis dissimilis* and other Lepidoptera. Unrooted tree was constructed using the BioNJ algorithm in Seaview v.4, which was made based on a sequence alignment using ClustalX 1.83. ORCO and PR genes are labeled in blue and red, respectively. Adis, *Athetis dissimilis*; Dple, *Danaus plexippus*; Cpom, *Cydia pomonella*; Bmor, *Bombyx mori*; Harm, *Helicoverpa armigera*.

A total of 73 different sequences that encode candidate OR genes were identified by bioinformatic analysis. Among them, 59 were deposited in the GenBank database under accession numbers in between KR935700 to KR935758, the one Orco gene was deposited under the accession number KR632987. Although 13 other sequences are either shorter than 200 bp or have no common sites found for computing distances, we did not exclude the possibility that they may represent non-conserved portions of genes. Hereby we only analyzed the 60 OR sequences used in our phylogenetic tree construction. The information on the 60 ORs can be found in **Table 4**, while the sequences of 13 residues OR gene were listed in **S2 File**. Confirmation was made by phylogenetic analysis for the four candidates AdisPR genes (AdisOR3, 6, 11, and 14), which clusters them into the conserved clade of lepidopteran species PRs. As expected, the AdisOrco sequence showed high homology to the conserved insect co-receptor clustered in the Orco clade. Aside from AdisOR47, all putative AdisORs were assigned to Lepidoptera ORs ortholog clades (**Fig 5**).

**Table 4:** Unigenes of candidate olfactory receptors.

Unigene reference	Gene name	ORF (aa)	BLASTx best hit (Reference/Name/Species)	E-value	Identify	Full length	TMD (No)	accession numbers
**Olfactory co-receptor**								
c73355.graph_c0	AdisOrco	473	dbj|BAG71415.1| olfactory receptor-2 [Mythimna separata]	8e-128	97%	Yes	7	KR632987
**Pheromone receptors**								
c64879.graph_c0	AdisOR3	435	gb|AGH58122.1| odorant receptor 16 [Spodoptera exigua]	5e-177	69%	Yes	6	KR935700
c68561.graph_c0	AdisOR6	129	gb|AGI96751.1| olfactory receptor 16 [Spodoptera litura]	2e-122	49%	No	0	KR935702
c71431.graph_c0	AdisOR11	442	gb|ACF32965.1| olfactory receptor 11 [Helicoverpa armigera]	0.0	76%	Yes	8	KR935707
c73498.graph_c0	AdisOR14	442	dbj|BAG71414.1| olfactory receptor-1 [Mythimna separata]	0.0	96%	Yes	5	KR935711
**Other olfactory receptors**								
c71708.graph_c1	AdisOR4	357	ref|NP_001116817.1| olfactory receptor-like [Bombyx mori]	3e-114	64%	Yes	5	KR935701
c74206.graph_c0	AdisOR7	406	gb|AGK90001.1| olfactory receptor 7 [Helicoverpa armigera]	0.0	83%	Yes	5	KR935703
c70218.graph_c0	AdisOR8	382	emb|CAD31949.1| putative chemosensory receptor 8 [Heliothis virescens]	8e-136	59%	Yes	6	KR935704
c62603.graph_c0	AdisOR9	203	gb|AGK90002.1| olfactory receptor 9 [Helicoverpa armigera]	4e-124	53%	Yes	3	KR935705
c68869.graph_c0	AdisOR10	383	gb|AGK90003.1| olfactory receptor 10 [Helicoverpa armigera]	0.0	90%	No	4	KR935706
c68437.graph_c0	AdisOR12a	456	gb|AGG08878.1| putative olfactory receptor 12 [Spodoptera litura]	0.0	68%	Yes	5	KR935708
c585.graph_c0	AdisOR12b	117	gb|AFC91721.1| putative odorant receptor OR12 [Cydia pomonella]	4e-21	53%	No	2	KR935709
c71384.graph_c0	AdisOR13	431	emb|CAG38113.1| putative chemosensory receptor 12 [Heliothis virescens]	0.0	80%	No	6	KR935710
c69788.graph_c0	AdisOR15	390	tpg|DAA05974.1| odorant receptor 15 [Bombyx mori]	5e-118	54%	Yes	6	KR935712
c67162.graph_c0	AdisOR16	388	ref|NP_001104832.2| olfactory receptor 16 [Bombyx mori]	3e-157	68%	Yes	6	KR935713
c61610.graph_c0	AdisOR17	393	gb|AFC91725.1| putative odorant receptor OR17 [Cydia pomonella]	1e-84	49%	Yes	6	KR935714
c69146.graph_c0	AdisOR18	398	gb|ACL81188.1| putative olfactory receptor 18 [Mamestra brassicae]	0.0	83%	Yes	5	KR935715
c56910.graph_c0	AdisOR19	402	gb|AGG08879.1| putative olfactory receptor 19 [Spodoptera litura]	4e-142	61%	Yes	6	KR935716
c69267.graph_c1	AdisOR20	392	gb|AGK90009.1| olfactory receptor 20 [Helicoverpa armigera]	0.0	78%	Yes	7	KR935717
c68838.graph_c0	AdisOR21	401	emb|CUQ99410.1| olfactory receptor 29 [Manduca sexta]	0.0	69%	Yes	6	KR935718
c75449.graph_c0	AdisOR22	316	dbj|BAR43488.1| putative olfactory receptor 46 [Ostrinia furnacalis]	3e-88	42%	No	4	KR935719
c49866.graph_c0	AdisOR23	114	gb|AFC91730.1| putative odorant receptor OR22, partial [Cydia pomonella]	2e-06	31%	No	2	KR935720
c69916.graph_c0	AdisOR24	321	gb|AFC91732.1| putative odorant receptor OR24 [Cydia pomonella]	3e-75	47%	Yes	4	KR935721
c54998.graph_c0	AdisOR25	421	dbj|BAH66322.1| olfactory receptor [Bombyx mori]	2e-86	42%	Yes	6	KR935722
c75146.graph_c0	AdisOR26	326	ref|NP_001091790.1| candidate olfactory receptor [Bombyx mori]	3e-154	67%	Yes	4	KR935723
c66614.graph_c0	AdisOR27	376	ref|NP_001166893.1| olfactory receptor 27 [Bombyx mori]	2e-111	65%	Yes	6	KR935724
c67713.graph_c0	AdisOR28	400	ref|NP_001166605.1| olfactory receptor 20 [Bombyx mori]	5e-115	52%	Yes	7	KR935725
c66566.graph_c0	AdisOR29	398	ref|NP_001166894.1| olfactory receptor 29 [Bombyx mori]	2e-161	68%	Yes	6	KR935726
c61198.graph_c0	AdisOR30	396	tpg|DAA05986.1| odorant receptor 30 [Bombyx mori]	1e-125	57%	Yes	5	KR935727
c70461.graph_c0	AdisOR31	407	gb|AGG08876.1| putative olfactory receptor 51 [Spodoptera litura]	0.0	85%	Yes	4	KR935728
c70212.graph_c0	AdisOR32a	87	dbj|BAG12812.1| olfactory receptor-like receptor [Bombyx mori]	4e-82	39%	No	0	KR935729
c61091.graph_c0	AdisOR32b	179	gb|AFC91741.1| putative odorant receptor OR33, partial [Cydia pomonella]	5e-42	49%	No	0	KR935730
c69561.graph_c0	AdisOR33	377	ref|NP_001103623.1| olfactory receptor 33 [Bombyx mori]	3e-87	40%	Yes	2	KR935731
c67193.graph_c0	AdisOR34	390	ref|NP_001103623.1| olfactory receptor 33 [Bombyx mori]	2e-77	34%	No	4	KR935732
c66964.graph_c1	AdisOR35	289	ref|NP_001166892.1| olfactory receptor 36 [Bombyx mori]	6e-101	60%	Yes	3	KR935733
c74970.graph_c0	AdisOR36	307	gb|AFL70813.1| odorant receptor 50, partial [Manduca sexta]	3e-112	58%	No	4	KR935734
c71270.graph_c0	AdisOR37	415	gb|AFL70813.1| odorant receptor 50, partial [Manduca sexta]	8e-122	53%	Yes	6	KR935735
c72094.graph_c0	AdisOR38	419	ref|NP_001103476.1| olfactory receptor 35 [Bombyx mori]	8e-133	58%	Yes	5	KR935736
c59825.graph_c0	AdisOR39	168	gb|AFL70813.1| odorant receptor 50, partial [Manduca sexta]	5e-45	54%	No	1	KR935737
c67128.graph_c1	AdisOR40	406	ref|XP_004925617.1| putative odorant receptor 85c-like [Bombyx mori]	2e-47	38%	Yes	7	KR935738
c72958.graph_c0	AdisOR41	392	ref|NP_001091818.1| olfactory receptor 42 [Bombyx mori]	1e-133	60%	Yes	7	KR935739
c61041.graph_c0	AdisOR42	173	ref|NP_001091818.1| olfactory receptor 42 [Bombyx mori]	8e-91	56%	No	4	KR935740
c74000.graph_c0	AdisOR43	252	ref|XP_004928758.1| putative odorant receptor 85c-like [Bombyx mori]	0.0	73%	Yes	2	KR935741
c67839.graph_c0	AdisOR44	429	gb|AGG08877.1| putative olfactory receptor 44 [Spodoptera litura]	0.0	90%	Yes	6	KR935742
c68687.graph_c0	AdisOR45	412	gb|AEF32141.1| odorant receptor [Spodoptera exigua]	0.0	82%	Yes	7	KR935743
c71141.graph_c0	AdisOR46	357	gb|AGK89999.1| olfactory receptor 3 [Helicoverpa armigera]	0.0	86%	Yes	6	KR935744
c69399.graph_c0	AdisOR47	393	ref|XP_003691419.1| odorant receptor 43a-like [Apis florea]	7e-08	24%	No	6	KR935745
c64283.graph_c0	AdisOR53	403	gb|AFC91736.1| putative odorant receptor OR28 [Cydia pomonella]	4e-138	55%	Yes	6	KR935746
c63838.graph_c0	AdisOR54	289	gb|EHJ72218.1| olfactory receptor-like receptor [Danaus plexippus]	2e-110	56%	Yes	5	KR935747
c69790.graph_c0	AdisOR56	402	ref|NP_001166617.1| olfactory receptor 56 [Bombyx mori]	7e-167	72%	No	5	KR935748
c66369.graph_c0	AdisOR58	228	gb|AGK90020.1| olfactory receptor 17 [Helicoverpa assulta]	6e-128	68%	Yes	4	KR935749
c64283.graph_c0	AdisOR59	107	gb|AFC91736.1| putative odorant receptor OR28 [Cydia pomonella]	4e-138	55%	No	0	KR935750
c73884.graph_c0	AdisOR60	230	ref|NP_001155301.1| olfactory receptor 60 [Bombyx mori]	0.0	70%	Yes	3	KR935751
c67100.graph_c0	AdisOR61	233	ref|XP_004932762.1| gustatory and odorant receptor 22-like [Bombyx mori]	1e-148	88%	Yes	3	KR935752
c65203.graph_c0	AdisOR62	395	ref|NP_001166603.1| olfactory receptor 13 [Bombyx mori]	2e-109	49%	Yes	6	KR935753
c67678.graph_c0	AdisOR63	391	ref|NP_001166620.1| olfactory receptor 63 [Bombyx mori]	4e-140	64%	No	5	KR935754
c71543.graph_c0	AdisOR64	420	ref|NP_001166621.1| olfactory receptor 64 [Bombyx mori]	2e-72	52%	No	4	KR935755
c69646.graph_c0	AdisOR65	419	ref|NP_001166621.1| olfactory receptor 64 [Bombyx mori]	6e-79	56%	No	6	KR935756
c66297.graph_c0	AdisOR66	225	emb|CAG38122.1| putative chemosensory receptor 21 [Heliothis virescens]	1e-97	75%	Yes	4	KR935757
c62997.graph_c0	AdisOR85	395	ref|XP_004925617.1| putative odorant receptor 85c-like [Bombyx mori]	7e-80	54%	Yes	6	KR935758

**Fig 6:**
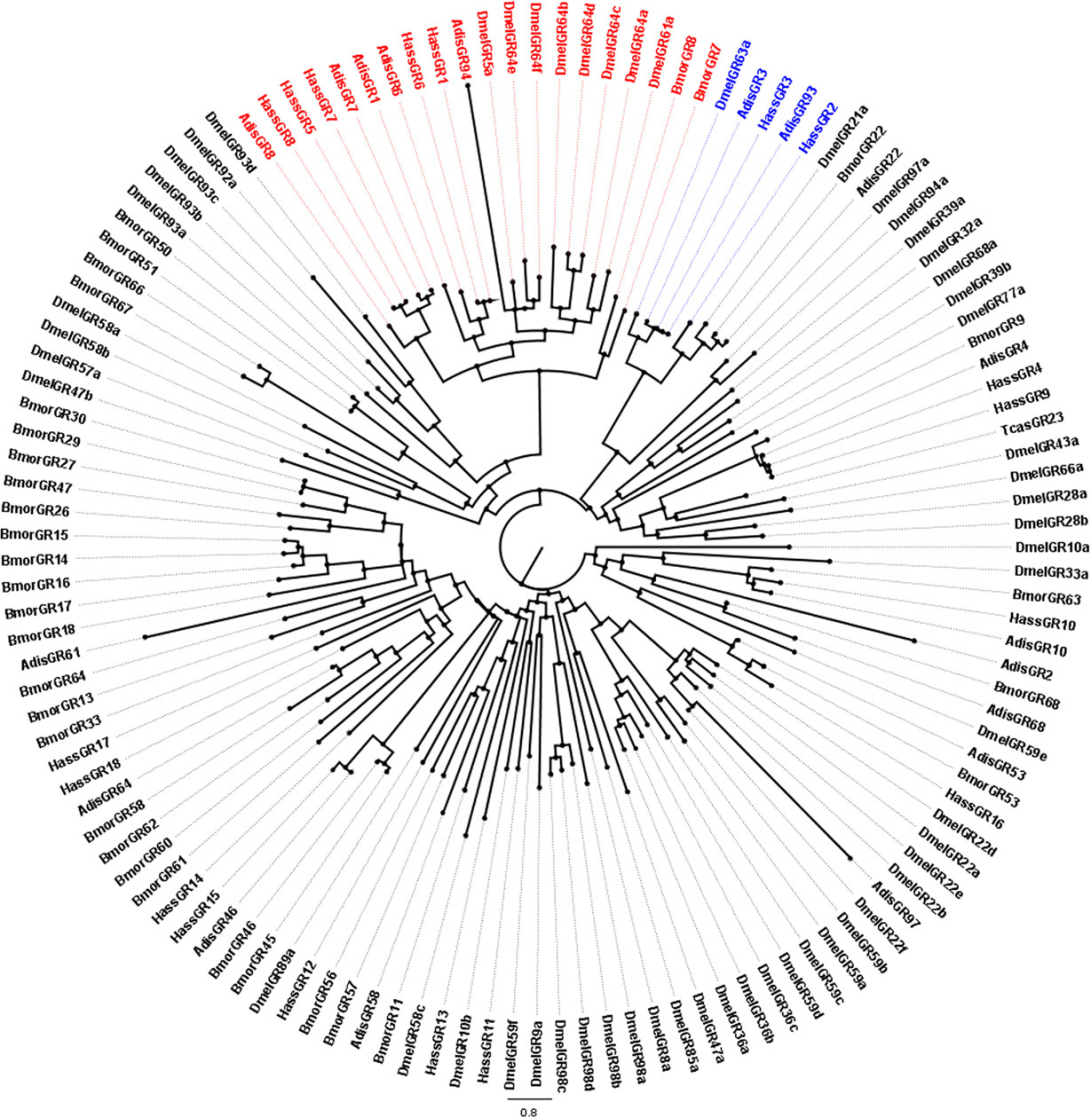
Neighbor-joining tree of candidate gustatory receptor (GR) genes from *Athetis dissimilis* and other insects. Unrooted tree was constructed using the BioNJ algorithm in Seaview v.4, which was made based on a sequence alignment using ClustalX 1.83. The red and blue indicate sugar and CO_2_ receptor genes, respectively. Adis, *Athetis dissimilis*; Dmel, *Drosophila melanogaster*; Bmor, *Bombyx mori*; Hass, *Helicoverpa assulta*.

In the current study, 18 candidate GRs from the *A*. *dissimilis* antennal transcriptome were identified. Only two GR genes were full-length ORFs while the others were only partial sequences. All these genes were registered to NCBI GenBank (KR674128-KR674145). The information on the GR genes was listed in **Table 5**. A phylogenetic tree was constructed using 18 candidate ApisGRs, 18 *H*. *assulta* GRs, 33 *B*. *mori* GRs, and 56 *D*. *melanogaster* GRs (**Fig 6**). AdisGR1, 6, 7, 8 and 94 are the members of the “sugar” receptor subfamily and they were classified as a clade with *H*. *assulta* “sugar” receptors (HassGR6, HassGR7 and HassGR8). In addition, two putative GR receptors (AdisGR3 and 93) were identified as the “CO_2_” receptor genes of the insect that are sharing high sequence identity with *H*. *assulta* “CO_2_” receptors (HassGR2 and HassGR3).

**Table 5:** Unigenes of candidate gustatory receptors.

Unigene reference	Gene name	ORF (aa)	BLASTx best hit (Reference/Name/Species)	E-value	Identify	Full length	TMD (No)	accession numbers
c51995.graph_c0	AdisGR1	80	gb|AIG51911.1| gustatory receptor [Helicoverpa armigera]	1e-37	86%	No	1	KR674128
c58414.graph_c0	AdisGR2	70	tpg|DAA06395.1| gustatory receptor 63 [Bombyx mori]	7e-93	30%	No	0	KR674129
c80317.graph_c0	AdisGR3	201	gb|EHJ78216.1| gustatory receptor 24 [Danaus plexippus]	1e-69	74%	No	3	KR674130
c67557.graph_c0	AdisGR4	164	gb|AGK90024.1| gustatory receptor 4 [Helicoverpa assulta]	1e-72	91%	No	2	KR674131
c54401.graph_c0	AdisGR6	266	gb|AGK90010.1| gustatory receptor 1 [Helicoverpa armigera]	2e-63	45%	No	2	KR674132
c68781.graph_c0	AdisGR7	429	gb|AGK90012.1| gustatory receptor 5 [Helicoverpa armigera]	2e-163	66%	Yes	6	KR674133
c64495.graph_c0	AdisGR8	348	ref|XP_004923090.1| putative gustatory receptor 64a-like [Bombyx mori]	5e-102	54%	Yes	6	KR674134
c10749.graph_c0	AdisGR10	229	tpg|DAA06395.1| gustatory receptor 63 [Bombyx mori]	2e-62	45%	No	4	KR674135
c80494.graph_c0	AdisGR22	185	ref|XP_004932762.1| gustatory and odorant receptor 22-like [Bombyx mori]	6e-93	76%	No	3	KR674136
c101589.graph_c0	AdisGR46	68	gb|ACD85125.1| gustatory receptor 46, partial [Bombyx mori]	6e-10	39%	No	2	KR674137
c109192.graph_c0	AdisGR53	81	tpg|DAA06389.1| gustatory receptor 53 [Bombyx mori]	1e-22	59%	No	1	KR674138
c55668.graph_c0	AdisGR58	111	gb|AJD81603.1| gustatory receptor 10, partial [Helicoverpa assulta]	7e-07	29%	No	2	KR674139
c77716.graph_c0	AdisGR61	120	gb|EHJ69979.1|putative gustatory receptor candidate 59 [Danaus plexippus]	1e-25	64%	No	1	KR674140
c18632.graph_c0	AdisGR64	80	tpg|DAA06392.1| gustatory receptor 58 [Bombyx mori]	1e-07	35%	No	2	KR674141
c91868.graph_c0	AdisGR68	146	ref|NP_001233217.1| gustatory receptor 68 [Bombyx mori]	9e-30	60%	No	0	KR674142
c84147.graph_c0	AdisGR93	91	gb|AJD81596.1| gustatory receptor 3 [Helicoverpa assulta]	3e-56	99%	No	1	KR674143
c16232.graph_c0	AdisGR94	68	ref|XP_001866271.1| Gustatory receptor 64a [Culex quinquefasciatus]	3e-04	32%	No	0	KR674144
c102934.graph_c0	AdisGR97	92	ref|XP_004932263.1| gustatory and odorant receptor 22-like [Bombyx mori]	5e-38	86%	No	0	KR674145

**Fig 7:**
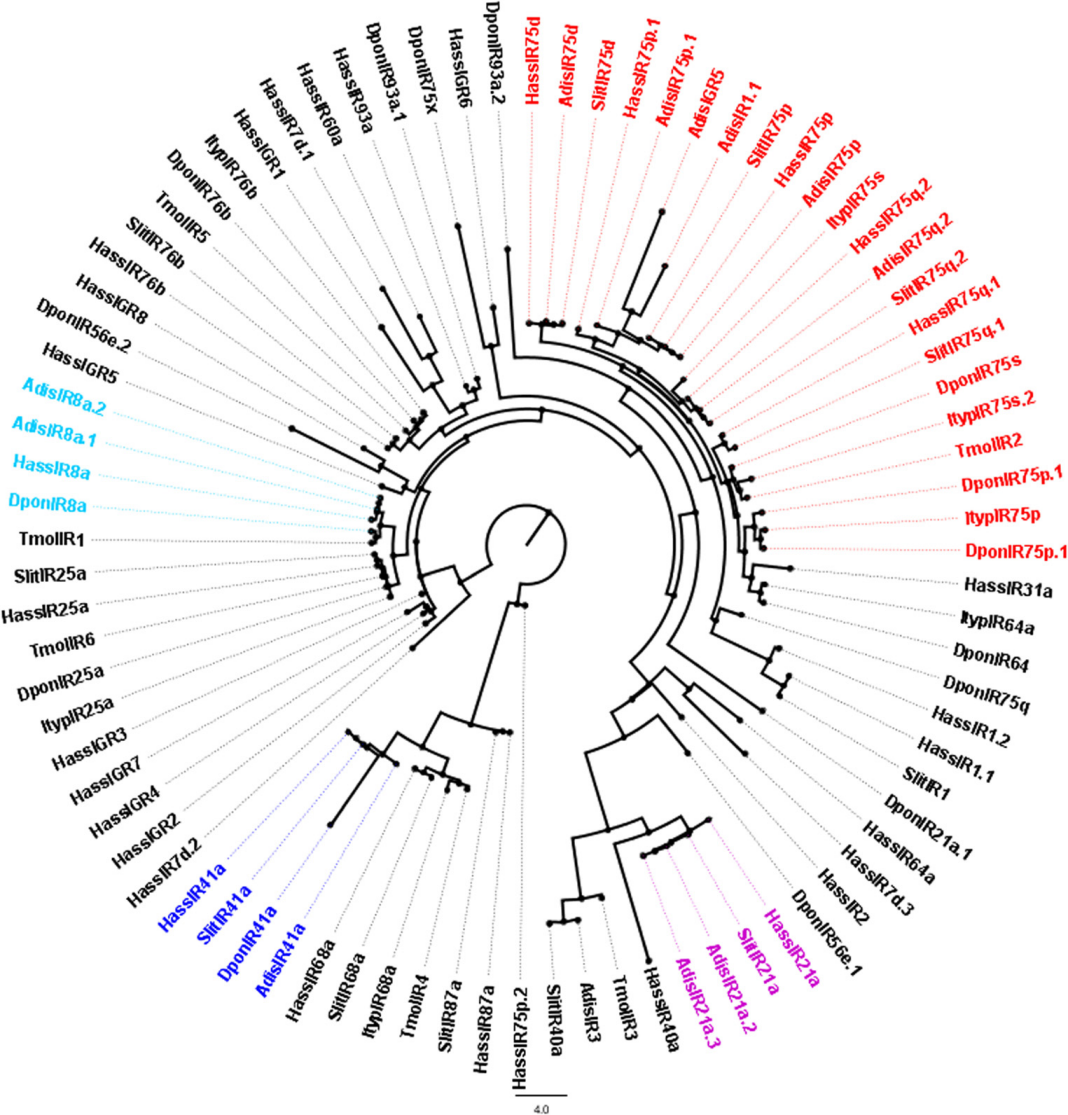
Neighbor-joining tree of candidate ionotropic receptor (IR) genes from *Athetis dissimilis* and other insects. Unrooted tree was constructed using the BioNJ algorithm in Seaview v.4, which was made based on a sequence alignment using ClustalX 1.83. Adis, *Athetis dissimilis*; Slit, *Spodoptera littoralis*; Hass, *Helicoverpa assulta*; Tmol, *Tenebrio molitor*; Dpon, *Dendroctonus ponderosae*; Ityp, *Ips typographus*.

We also identified 12 candidate IR genes according to their similarities to known insect IRs, in which 4 sequences with full-length ORFs and 8 sequences with incomplete 5′ or 3′ terminus. These 12 sequences were deposited in the GenBank under succeeding accession numbers from KR912012 to KR912023. The information on the IRs was listed in **Table 6**. *A*. *dissimilis *IRs were named for their homology to those of *H*. *assulta* and *S*. *littoralis*. AdisIR8a.1 and 8a.2 were phylogenetically clustered with the highly conserved IR8a sub-family, but no single IR gene of *A*. *dissimilis* was located in the IR25a sub-family. Two IRs were clustered into the SlitIR1/HassIR1.1 clade, with reliable bootsrap support, named as AdisIR1.1 and 1.2. IR75 was a very large clade that comprises four *A*. *dissimilis* IRs (AdisIR75d, 75q.2, 75p and 75p.1). Further, IR21a (containing Adis21a.2 and 21a.3) and IR41a (containing Adis41a) were also highly conserved clades. At least one insect IR orthologous could be assigned to the majority of the putative AdisIRs (**Fig 7**).

**Table 6:** Unigenes of candidate ionotropic receptors.

Unigene reference	Gene name	ORF (aa)	BLASTx best hit (Reference/Name/Species)	E-value	Identify	Full length	TMD (No)	accession numbers
c75167.graph_c1	AdisIR75p.1	434	gb|ADR64684.1|putative chemosensory ionotropic receptor IR75p [Spodoptera littoralis]	0.0	85%	No	2	KR912012
c73367.graph_c2	AdisIR75q.2	627	gb|ADR64685.1|putative chemosensory ionotropic receptor IR75q.2 [Spodoptera littoralis]	0.0	78%	Yes	4	KR912013
c57740.graph_c0	AdisIR21a.2	76	gb|ADR64678.1|putative chemosensory ionotropic receptor IR21a [Spodoptera littoralis]	5–33	88%	No	0	KR912014
c70360.graph_c0	AdisIR41a	474	gb|ADR64681.1|putative chemosensory ionotropic receptor IR41a [Spodoptera littoralis]	0.0	77%	Yes	3	KR912015
c79758.graph_c0	AdisIGR5	66	gb|AFC91759.1|putative ionotropic receptor iGluR, partial [Cydia pomonella]	1e-21	90%	No	0	KR912016
c69917.graph_c0	AdisIR75d	601	gb|ADR64683.1|putative chemosensory ionotropic receptor IR75d [Spodoptera littoralis]	0.0	74%	Yes	3	KR912017
c75352.graph_c0	AdisIR8a.1	896	gb|AFC91764.1|putative ionotropic receptor IR8a, partial [Cydia pomonella]	0.0	78%	Yes	3	KR912018
c75366.graph_c0	AdisIR21a.3	853	gb|ADR64678.1|putative chemosensory ionotropic receptor IR21a [Spodoptera littoralis]	0.0	81%	No	3	KR912019
c68537.graph_c0	AdisIR75p	222	gb|ADR64684.1|putative chemosensory ionotropic receptor IR75p [Spodoptera littoralis]	2.08851e-105	87%	No	1	KR912020
c60783.graph_c0	AdisIR3	255	gb|ADR64680.1|putative chemosensory ionotropic receptor IR40a [Spodoptera littoralis]	1e-108	91%	No	1	KR912021
c48687.graph_c0	AdisIR8a.2	67	gb|AFC91764.1|putative ionotropic receptor IR8a, partial [Cydia pomonella]	5e-13	64%	No	0	KR912022
c59371.graph_c0	AdisIR1.1	100	gb|ADR64688.1|putative chemosensory ionotropic receptor IR1 [Spodoptera littoralis]	1e-14	53%	No	0	KR912023

To analysis the transcription abundance of global chemosensory receptor genes in the sequenced libraries of both sexes, we surveyed the differential expression of all chemosensory receptor ORFs identified in the present study. The result is listed in **S3 File**.

## Discussion

Transcriptome sequencing is a feasible and economical way to obtain target genes of interest in a short time; this technology has become popular for filtering chemosensory receptors from insect antennae transcriptome. This has been accomplished for the orders and relevant species like: Lepidoptera: *Manduca sexta* (47 ORs, 6 IRs) [35], *H*. *armigera* (47 ORs, 12 IRs) [7], *C*. *pomonella* (43 ORs, 15 IRs) [23]; Hymenoptera: *Nasonia vitripennis* (225 ORs, 58 GRs) [36], *Apis mellifera* (170 ORs, 10 GRs) [37], *Glossina morsitans morsitans* (46 ORs, 14 GRs) [38]; Coleoptera: *Megacyllene caryae* (57 ORs) [39], *T*. *molitor* (20 ORs, 6 IRs) [32]; Diptera: *Calliphora stygia* (50 ORs, 21GRs, 22 IRs) [24], *Anopheles gambiae* (75 ORs, 61 GRs, 46 IRs) [40]; and Homoptera: *Aphis gossypii* (45 ORs, 14 IRs) [41]; and Orthoptera: *Locusta migratoria* (142 ORs, 32 IRs) [42]. The genus *Athetis* is a group of 211 species [43]. Although the majority of the species are not considered as insect pests with major economic effects, a few *Athetis* species such as *A*. *lepigone*, *A*. *dissimilis* and *A*. *gluteosa* are identified as important crop pest insects in China. Here, we identified 60 candidate OR gene sequences, 18 GRs and 12 IRs from *A*. *dissimilis*. This is the first report in the genus *Athetis*, to our knowledge, that the olfactory receptors of moths are identified by using transcriptome technology with a transcriptome strategy proved to be effective in uncovering large sets of chemoreceptor from three major gene families.

ORs, sex-biased expression in the antennae of one sex, are generally considered as PRs that mediate behaviors specific to that sex. Lepidoptera sex pheromones produced by females may attract males for mating opportunities. Several moth sex pheromone receptors have now been functionally characterized, and most are expressed at higher levels in the male antennae [44–46]. Based on phylogenetic tree analyzes of the *A*. *dissimilis* ORs, four of them clustered in a conserved clade of PRs found in Lepidopteran insects (**Fig 5**). We, therefore, hypothesize that some or all of them appear to be dedicated to sex pheromone detection. Accordingly, results from the transcription abundance analysis (**S3 File**) showed that *AdisOR3*, *6* and *14* had very high expression quantities in the male antennae, while the gene expression level of *AdisOR11* was the only one that is almost equal in the female and male antennae. *AdisOR11* showed equal expression levels in male and female antennae, which may relate to females detecting their own pheromones.

Insect ORs are frequently co-expressed with a nonconventional OR, recently renamed as olfactory receptor co-receptor (Orco) while they were previously referred to as OR83b in *D*. *melanogaster* and OR2 in *B*. *mori* [47]. Unlike other insect ORs, with a little sequence homology, Orco is strikingly well conserved across insect species. We identified one AdisOrco sequence with a high degree of similarity to co-receptors from different insect orders clustered in the Orco clade. We found that the *AdisOrco* gene with biased male expression has the highest expression quantity in all OR genes from the female and male adult antennae (Please see **S3 File**). This is also in accordance with the expression pattern of all insect Orco genes.

The GR family of insect chemoreceptors includes receptors for sugars and bitter compounds, as well as cuticular hydrocarbons and odorants such as CO_2_. Gustatory receptors perceive essential nutrients whose chemical structures remain constant (compared to bitter-tasting, secondary plant compounds) such as sugars and CO_2_ receptors. Thus, sugar and CO_2_ receptor genes are relatively highly conserved in most of the insect genomes that have been sequenced to date [10,24,29,48]. We have annotated 18 GR genes from the *A*. *dissimilis* antennal transcriptome dataset. The GR family in *A*. *dissimilis* includes two putative CO_2_ receptors (AdisGR3 and 93) and five sugar receptors (AdisGR1, 6, 7, 8 and 94). AdisGR4, the putative gustatory receptor, share the same clade with BmorGR9, HassGR4 and HassGR9. In comparison with BmorGR9, a newly characterized receptor of fructose [49], we can suggest that AdisGR4 is a sugar receptor (**Fig 6**). Sugars and sugar alcohols have been identified to affect the host plant selection and egg-laying behavior of codling moth females [50].

The iGluRs mediate excitatory neurotransmission in both vertebrate and invertebrate nervous systems [51]. Ionotropic receptor genes were first discovered in *D*. *melanogaster* through genome analyzes [13]; they arose from an iGluR with a change in expression localization from an interneuron to a sensilla neuron [22]. In *D*. *melanogaster* antennae, IRs have been reported to detect a variety of molecules [52]. In the *A*. *dissimilis* antennal transcriptome, we identified 11 candidate IRs and 1 candidate iGluR. Recent studies have indicated that the coreceptors of IRs, *IR8a/25a* have a similar expression pattern with the *Orco*, playing an essential role in tuning IRs sensory cilia targeting and IR-based sensory channels [52]. Although we identified two IR8a genes from *A*. *dissimilis* namely AdisIR8a.1 and AdisIR8a.2, IR25a was not found. This may be the result of no biological repeats. We also found the expression level of *AdisIR8a*.*1* was rather high and analogous to the *Orco* (Please see **S3 File**). While two IRs named AdisIR1.1 and 1.2 clustered together with their orthologous SlitIR1/HassIR1.1 in a “divergent IR” clade, four IRs (AdisIR75d, 75q.2, 75p, and 75p.1) are localized in a large clade of IR75. But so far, the function of IR75 is unclear. Moreover, IR21a (containing Adis21a.2 and 21a.3), IR76b and IR41a (containing Adis41a) were also highly conserved clades. All AdisIRs that we discovered have orthologs found in Hass/Slit/Dpon.

## Conclusions

We first obtained abundant biology information on the transcriptome of *A*. *dissimilis* antennae using high-throughput sequencing technology with the aim of identifying of the genes potentially involved in the olfaction process. From the obtained transcriptome data, three important gene families encoding chemosensory receptors were identified, annotated, and further analyzed for their expression profile. Our results provide a foundational knowledge for exploring and understanding the molecule mechanism involved in olfactory recognition process of the insect pest *A*. *dissimilis*, and providing alternative novel targets for the pest management with semiochemicals.

## Supporting Information

S1 File Amino acid sequences of ORs, GRs and IRs were used in phylogenetice analyses. (TXT)
(TXT)

S2 File The amino acid sequences of 13 candidate olfactory genes identified in this study were not used in phylogenetice analyses. (TXT)
(TXT)

S3 File Comparison of expression of ORs, GRs and IRs in female and male antennae as revealed by mapping Illumina read. (DOC)
(DOCX)
