# Supplementary material for: Identification of Putative Chemosensory Receptor Genes from the Athetis dissimilis Antennal Transcriptome
Source: PLoS One. 2016 Jan 26;11(1):e0147768. doi: 10.1371/journal.pone.0147768 (PMC4727905; doi:10.1371/journal.pone.0147768)
Supplement: S3 File — (DOC) (DOCX) [file pone.0147768.s003.docx]

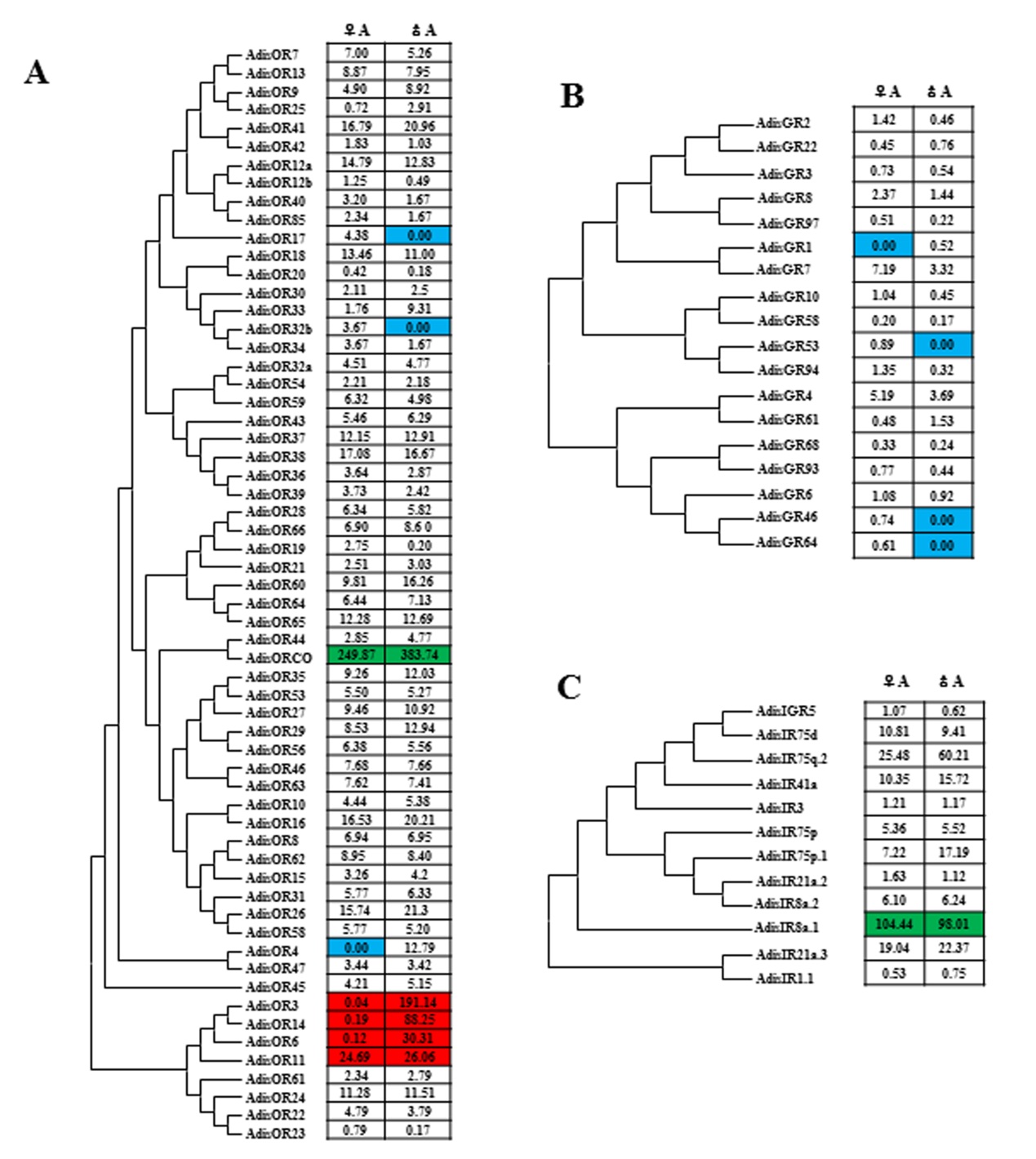


S3 Fig. Comparison of expression of (A) olfactory receptor (OR), (B) gustatory receptor (GR) and (C) ionotropic receptor (IR) genes in female and male antennae as revealed by Illumina read mapping. In each box, the relative abundance value in Fragments Per Kilobase of transcript per million mapped reads (FPKM) of each receptor gene is indicated. The candidate pheromone receptors are labeled in red. Green indicates the genes of high expression, and blue indicates the genes of low expression. **♀A**: female antennae, **♂A**: male antennae.
